# Working hours, on‐call shifts, and risk of occupational injuries among hospital physicians: A case‐crossover study

**DOI:** 10.1002/1348-9585.12322

**Published:** 2022-03-17

**Authors:** Annina Ropponen, Aki Koskinen, Sampsa Puttonen, Jenni Ervasti, Mika Kivimäki, Tuula Oksanen, Mikko Härmä, Kati Karhula

**Affiliations:** ^1^ 3860 Finnish Institute of Occupational Health Helsinki Finland; ^2^ Division of Insurance Medicine Department of Clinical Neuroscience Karolinska Institutet Stockholm Sweden; ^3^ Clinicum Faculty of Medicine University of Helsinki Helsinki Finland; ^4^ Department of Epidemiology and Public Health University College London London United Kingdom; ^5^ School of Medicine Institute of Public Health and Clinical Nutrition University of Eastern Finland Kuopio Finland

**Keywords:** injury, on‐call work, physician, shift work, working hours

## Abstract

**Objective:**

To investigate the association of hospital physicians’ working hours and on‐call shifts with the risk of occupational injuries.

**Methods:**

In this nested cohort study of 556 Finnish hospital physicians, we linked electronic records from working‐hour and on‐call duty payroll data to occupational injury data obtained from the Finnish Workers’ Compensation Center for the period 2005–2019. We used a case‐crossover design with matched intervals for a 7‐day ‘case window’ immediately prior to occupational injury and a ‘control window’ 7 days prior to the beginning of the case window, and analyzed their associations using conditional logistic regression models.

**Results:**

We noted 556 occupational injuries, 281 at the workplace and 275 while commuting. Having three to four long (>12 h) work shifts on the preceding 7 days was associated with a higher probability of an occupational injury (odds ratio [OR] 2.14, 95% confidence interval [CI] 1.11, 4.09), and the OR for three to four on‐call shifts was 3.54 (95%CI 2.11, 5.92) in comparison to having none of these work shift types. A higher number of several consecutive working days was associated with a higher probability of injury in a dose‐response manner. Moreover, increasing weekly working hours was associated with an increased likelihood of injury (OR 1.03, 95%CI 1.01, 1.04), whereas the number of normal (≤12 h) work shifts reduced this likelihood (OR 0.79, 95%CI 0.64, 0.98).

**Conclusions:**

Our findings suggest that accumulated working‐hour load, as opposed to single, very long (>24 h) work shifts, may increase the risk of occupational injury among hospital physicians.

## 1 INTRODUCTION

To supply 24/7 patient care at hospitals, physicians’ work is arranged as irregular working hours with varying start and finish times, shift lengths, and recovery periods between shifts.^1^ Of the hospital physicians in the study, at least 50% worked on‐call hours,^2^, ^3^ and many also worked extra hours in addition to their normal daily working hours.^4^ On‐call duties, which often include extended working hours, consist of a high work pace due to the demands of care.^1^, ^4^ Earlier studies have indicated that on‐call work is associated with several negative health outcomes, such as work stress, burnout, and occupational injuries.^5^, ^6^ The mechanisms linking physicians’ extended working hours and insufficient recovery to safety, health, and performance are well characterized.^7^ However, it remains unclear whether extended daily and weekly hours reduce the safety of the physicians themselves.^8^ Hence, there is an emergent need to identify the working‐hour characteristics that are associated with health and safety risks and to provide justifications for working‐hour restrictions for hospital physicians.

Previous studies of physicians’ working hours and on‐call work have utilized self‐reported data, which are prone to recall or reporting bias.^9^ Further limitations include the use of cross‐sectional designs^10^ and study populations that cover only a few medical specialties. Recently, objective and detailed daily working‐hour data from employers’ registries have become available for studies of health care personnel,^11^ although few studies to date have investigated the association between extended working hours and safety among physicians.^12^ Further drawbacks related to research on occupational injuries are the use of self‐reported data on working hours^13^ and a limited focus on, for example, needle‐stick injuries only.^14^ Further, existing register studies of physicians’ injuries have not used working‐hour data.^15^ Therefore, a study utilizing registry‐based working‐hour characteristics linked with national register data of occupational injuries is valuable for examining the associations between working hours, on‐call work, and occupational injuries among hospital physicians.

## 2 MATERIALS AND METHODS

### 2.1 Sample

Electronic payroll‐based working‐hour and on‐call work data were available from the records of seven Finnish hospital districts. The full daily working‐hour and on‐call work data were linked with occupational injury data obtained from the Finnish Workers’ Compensation Center, which had over 15 million observations from 14 704 physicians. The data from January 1, 2005 to December 31, 2019 included information on the hospital district and the age and sex of the physicians. First, the data were restricted to physicians with registered occupational injuries, totaling 988 individuals. Second, the data were restricted to physicians who had working‐hour data (i.e., at least one work shift) for the 14 preceding days (19 821 observations). The final sample consisted of 556 physicians (64% women). We received permission to access the working‐hour and on‐call data and employment information (i.e., work unit), and the data on occupational injuries from the hospital districts participating in the study. Since these data comprised register‐based employer‐owned employment information and no health information, ethical approval was not required for the study.

### 2.2 Occupational injuries

Occupational injuries were defined in accordance with the Finnish legislation (the Workers’ Compensation Act) used by the Workers’ Compensation Center (see https://www.tvk.fi/en/compensation/occupational‐accident/) that provided our data. Based on Finnish law, occupational injuries at the workplace also cover those that occur during activities other than work tasks and while commuting between the home and the workplace. The possible costs of all occupational injuries are reimbursed to the employer and the employees via a statutory insurance system (see in more detail^11^). The injury data include the date and place of the injury, (at work/while commuting/during leisure time) and the reported external cause, location, and type of injury (e.g., falling, crashes, violence, physical load).

### 2.3 Working‐hour characteristics and on‐call duties

Daily working‐hour and on‐call shift data (later working‐hour data) were retrieved from the Titania^®^ (CGI Finland Ltd) records on working‐hour characteristics without on‐call shifts, and the Titania^®^ for Physicians (CGI Finland Ltd) records on on‐call duties.^16^ The working‐hour data included actual payroll‐based start/end times of work and absences, including days off, sickness, and other leaves. Based on the generally used proxies for long working hours and time for recovery, we created the following measures for this study: weekly working hours (mean), shift length (mean), and number of work shifts of various lengths (≤12 h, >12 h, and >24 h). We also defined shift intensity using the number of consecutive work shifts and the number of short (< 11 h) shift intervals (i.e., the recovery time between the work shifts). Furthermore, we calculated the number of on‐call shifts. To categorize the thresholds, we utilized the cut‐off points based on the recommendations of the Finnish Institute of Occupational Health (FIOH) concerning consecutive working days and short (<11 h) shift intervals.

### 2.4 Matched interval case‐crossover design

This study applied a case‐crossover design with a matched‐paired interval approach, in which each physician with an occupational injury serves as their own control. Hence, there is a ‘case window’, i.e., the period for which the person is a case, and a ‘control window’, i.e., the period during which the physician is a non‐case (their own control). This design compares the exposure (working hours) during the case window to the exposure during the control window and each physician represents a matched set of data for both the case and control periods. The seven‐day case‐control time windows were set a priori. This design also controls for all the stable confounding factors, including time invariant covariates, such as sex, age, socioeconomic status, job titles, and organizational factors (e.g., workplace culture, management), which are all potential sources of confounding in other types of epidemiological designs, such as case‐control or cohort studies.

### 2.5 Statistical analyses

For the continuous variables, the descriptive characteristics of working hours and the differences between the 7‐day case and control windows were estimated using the t‐test. For the categorical variables, we used the Pearson's Chi‐squared test. We calculated the conditional logistic regression models with the case and control windows to estimate the probability of occupational injury, comparing the case window to the control window with odds ratios (OR) and 95% confidence intervals (95%CI). To control for the clustering effect, we used hospital district as a covariate in the analyses of all the working‐hour characteristics to adjust the standard errors. Stata MP version 17.0 (StataCorp., College Station, Texas, USA) was used for all the analyses.

## 3 RESULTS

In the final sample (*n* = 556), the mean age of the physicians was 41.3 years (SD 10.1, range 21–68 years). A total of 276 occupational injuries occurred at the workplace, of which 51% were injuries to finger(s), and 7% to hand(s), most commonly cuts or superficial injuries (69%), or luxations, twists, or strains (14%). As many as 275 injuries took place while commuting, of which 22% were traumas on multiple body locations and 12% injuries to legs. The commuting injuries were most caused by luxations, twists, or strains (36%), or were cuts or superficial injuries (29%). Five injuries took place while the person was driving during work. Table 1 shows the descriptive characteristics and the tests of the differences in the 7‐day case and control windows of the final sample.

**TABLE 1:** Descriptive statistics (mean, standard deviation [SD]) and frequencies (with proportion, %) of working‐hour characteristics and on‐call shifts in 7‐day case and control windows

Working‐hour characteristics					*p*‐value for difference^b^
	Physicians, *n* = 556				
	Case window		Control window		
	Mean	SD	Mean	SD	
Length of working hours					
Weekly working hours (h)	23.8	13.5	21.8	14.1	.023
Shift length (h)	9.1	2.3	9.1	2.4	ns.
Number of normal (≤12 h) work shifts	6.3	1.2	6.4	1.2	ns.
Number of long (>12 h) work shifts	0.6	1.2	0.6	1.1	ns.
Number of very long (>24 h) work shifts	0	0	0	0	ns.
Shift intensity					
Number of consecutive working days	5.3	2.8	4.9	2.9	.032
Number of work shifts in a day	0.7	1.0	0.6	1.0	ns.
Number of on‐call shifts	0.9	1.5	0.7	1.3	ns.
Number of short (<11 h) shift intervals	1.7	1.0	1.7	1.1	ns.
Thresholds for working‐hour characteristics	*n*	%	*n*	%	
Number of long (>12 h) work shifts					ns.
0	345	62	373	66	
1–2	183	33	166	30	
3–4	16	3	11	2	
≥5	12	2	12	2	
Number of very long (>24 h) work shifts					ns.
0	546	98	550	98	
1–2^a^	10	2	10	2	
Shift intensity					
Number of consecutive working days					ns.
1–2	42	8	78	14	
3–4	89	17	78	14	
5–6	309	56	311	56	
7	95	18	89	16	
Number of work shifts in a day					ns.
1	374	68	389	69	
2	174	31	164	29	
3^a^	8	1	9	2	
Number of on‐call shifts					.055
0	309	56	336	60	
1–2	201	36	196	35	
3–4	27	5	11	2	
≥5	19	3	19	3	
Number of short shift intervals					ns.
0–1	200	36	211	38	
2–4^a^	356	64	349	62	

^a^ Due to no or very few (≤10) observations, further categories were collapsed to the previous category and not shown.

^b^ Differences in continuous variables were tested by the t‐test and in categorical variables by the Pearson's Chi‐squared test.

The ORs of the 7‐day case and control windows indicated that weekly working hours (1.03, 95% CI 1.01, 1.04), working three to four long (>12 h) work shifts (2.14, 95%CI 1.11, 4.09), or three to four on‐call shifts (3.54, 95%CI 2.11, 5.92) were associated with an increased likelihood of occupational injury. The number of normal (≤12 h) work shifts was associated with a decreased likelihood of injuries. The number of consecutive working days was associated with an increased likelihood in a dose‐response manner (Table 2). The number of short shift intervals lacked a statistically significant association with injuries.

**TABLE 2:** Conditional logistic regression (odds ratio, OR with 95% confidence intervals, CI) for associations between working‐hour characteristics and on‐call shifts in 7‐day case and control windows and occupational injuries of 556 physicians

	OR	95%CI
Length of working hours		
Weekly working hours (h)	**1.03**	**1.01, 1.04**
Shift length (h)	1.05	0.93, 1.19
Number of normal (≤12 h) work shifts	**0.79**	**0.64, 0.98**
Number of long (>12 h) work shifts (ref = 0)		
1–2	1.28	0.93, 1.78
3–4	**2.14**	**1.11, 4.09**
≥5	na	–
Number of very long (>24 h) work shifts (ref = 0)		
1–2	0.90	0.65, 1.24
Shift intensity		
Number of consecutive working days (ref = 1–2)		
3–4	**3.24**	**2.29, 4.60**
5–6	**3.23**	**2.18, 4.80**
7	**4.10**	**2.20, 7.63**
Number of work shifts in a day (ref = 1)		
2	1.18	0.97, 1.43
3	0.67	0.41, 1.08
Number of on‐call shifts (ref = 0)		
1–2	1.24	0.79, 1.94
3–4	**3.54**	**2.11, 5.92**
≥5	na	–
Number of short (<11 h) shift intervals (ref = 0–1)		
2–4	1.07	0.84, 1.37
≥5	1.07	0.08, 14.38

Note

na = too few observations for analysis. Statistically significant values are indicated in bold.

## 4 DISCUSSION

This case‐crossover analysis nested in a prospective cohort study used objective working‐hour records linked to national register data on occupational injuries to examine the risk of injuries associated with on‐call work and working‐hour characteristics among 556 physicians. Weekly working hours, three to four long (>12 h) work shifts, or three to four on‐call shifts were associated with an increased likelihood of occupational injuries. A higher number of consecutive working days was associated with an increased likelihood of injury in a dose‐response manner. In turn, the number of normal (≤12 h) work shifts was associated with a decreased likelihood.

This study adds to earlier research that has mainly used self‐reported data,^9^ cross‐sectional design,^10^ few medical specialties, prevalence of injuries,^15^ or only needle‐stick injuries. Our analysis, using 7‐day windows to estimate the likelihood of occupational injuries, suggested that shift intensity, as indicated by the number of consecutive working days and number of on‐call shifts, increased the likelihood of occupational injury. However, the number of very long (>24 h) work shifts or short (<11 h) shift intervals was not associated with an increased likelihood of injury.

Due to the relatively small sample size, these results should be interpreted with caution. It may be hypothesized that both long working periods (i.e., >12‐h work shifts) and the number of on‐call shifts need to be limited, as they might cause irregularity in the schedules of physicians. The finding that a higher number of consecutive working days were associated with an increased probability of injuries might indicate that shorter working periods with opportunities to recuperate in between might protect from injuries. Short shift intervals were prevalent among the physicians, due to on‐call work and irregular working hours. Further studies with even larger sample sizes are needed to investigate the role of short recovery time in the safety of physicians and their patients.

A strength of this study was its use of objective working‐hour data from employer's registers and national register data on occupational injuries. Register data are free from memory bias and have no (or minimal) loss to follow‐up. Another strength is the use of matched interval case‐crossover design which does not require a separate comparison group, i.e., selection bias is minimized.

The main limitation of the study was its relatively low number of physicians with occupational injuries. Due to the total of 556 injuries, we had insufficient statistical power to run separate analyses of men and women, or to investigate those with on‐call duties in greater detail. Further research is needed to examine whether, due to only a few actual events during follow‐up, our findings regarding hospital physicians are generalizable to hospital physicians in all European Union and other countries with similar total working hours.

## 5 CONCLUSIONS

Our findings, based on 7‐day analysis windows, suggest that accumulated working‐hour load, as opposed to single very long (>24 h) work shifts, may increase the risk of occupational injury among hospital physicians.

## DISCLOSURE

Ethical approval: N/A. The data comprised register‐based employer‐owned employment information with no health information, so no ethical approval was required for the study. Informed consent: N/A. Registry and the Registration No. of the study/Trial: N/A. Animal Studies: N/A. Conflict of interest: The authors declare no conflicts of interest for this article.

## AUTHOR CONTRIBUTIONS

A.R., M.H., K.K., S.P., M.K., and T.O. conceived the ideas; K.K. and A.K. collected the data; A.R. analyzed the data; A.R. led the writing, and all the authors critically commented on the manuscript and approved the final version.

## Data Availability

Research data are not shared.
